# MiR-148b-3p Regulates the Expression of DTYMK to Drive Hepatocellular Carcinoma Cell Proliferation and Metastasis

**DOI:** 10.3389/fonc.2021.625566

**Published:** 2021-12-24

**Authors:** Guifang He, Jing Qiu, Changchang Liu, Ben Tian, Duo Cai, Shihai Liu

**Affiliations:** ^1^ Medical Animal Laboratory, The Affiliated Hospital of Qingdao University, Qingdao, China; ^2^ Department of Stomatology, Qingdao Municipal Hospital, Qingdao, China; ^3^ Department of Neurosurgery Intensive Medicine, The First Affiliated Hospital of Baotou Medical College, Baotou, China

**Keywords:** DTYMK, hepatocellular carcinoma cell, miRNA-148b-3p, cell proliferation, metastasis

## Abstract

Deoxythymidilate kinase (DTYMK) has been identified as a putative oncogene associated with the incidence of hepatocellular carcinoma (HCC), but the mechanisms whereby it regulates this cancer type remain uncertain. The present study was therefore designed to explore the role of DTYMK in HCC and to evaluate the underlying molecular mechanisms. MiRNAs associated with DTYMK expression levels in HCC were identified through analyses of both clinical samples and publically available gene expression datasets. We then assessed the putative functions of DTYMK and miR-148b-3p in this oncogenic context through studies of HCC cells and a murine xenograft model system. Correlation analyses and *in vitro* experiments led us to confirm DTYMK as a target of miR-148b-3p. In addition, we assessed dTTP levels associated with the DTYMK pathway in HCC cells to understand the functional implications of our experimental findings. We found that HCC tissues and cells exhibited marked DTYMK upregulation and miR-148b-3p downregulation, with the expression levels of DTYMK and miR-148b-3p being negatively correlated with one another. The impact of overexpressing DTYMK in tumor cells was partially reversed upon cellular transfection with miR-148b-3p mimics, providing conclusive evidence that DTMYK is a target of this miRNA. Importantly, DTYMK-related dTTP levels were also impacted by miR-148b-3p mimic transfection. DTYMK is a key regulator of HCC progression, and its expression is suppressed by miR-148b-3p, suggesting that this miR-148b-3p/DTYMK regulatory axis may be amenable to therapeutic targeting in patients with HCC.

## Introduction

Hepatocellular carcinoma (HCC) is among the deadliest cancers globally, in addition to being the most prevalent form of liver cancer (1). While a number of studies have identified viable surgical approaches and therapeutic targets in those with HCC, patients with advanced-stage disease still have a very poor prognosis (2). This is primarily attributable to the complex genetic and epigenetic factors regulating the development and progression of HCC.

The silencing of deoxythymidilate kinase (DTYMK) in the context of LKB1 loss in LKB1/KRAS double mutant non-small cell lung cancer (NSCLC) has been shown to induce synthetic lethality (3). DTYMK is an enzyme responsible for catalyzing deoxythymidine monophosphate (dTMP) conversion into deoxythymidine diphosphate (dTDP), making it an essential regulator of nucleotide synthesis. Liu et al. determined that inhibiting DTYMK in NSCLC cells bearing LKB1 mutations ultimately results in the misincorporation of dUTP into DNA, thereby blocking DNA replication. There is additional evidence linking DTYMK to mitochondrial DNA depletion syndrome (4) and HCC patient prognosis (5).

MicroRNAs (miRNAs) are short (~20 nucleotides) RNAs that lack coding potential (6), but that are capable of binding to 3’-untranslated region (3’-UTR) sequences in target mRNAs so as to control their stability and translation, thereby regulating gene expression (7). Bioinformatics-based analyses suggest that roughly 60% of mRNAs in humans can be targeted by miRNAs (8, 9). These miRNAs can additionally interact with other species of non-coding RNAs such as long non-coding RNAs (lncRNAs) (10–12), thus allowing them to regulate diverse processes such as apoptosis, migration, and proliferation (13, 14). As they are often dysregulated in tumor cells, miRNAs can function to promote or suppress tumor growth through many different mechanisms (15, 16). To date, however, few studies have evaluated the role of miRNAs as regulators of DTYMK in the context of oncogenesis. The present study was therefore designed to explore the functional relevance of DTYMK in HCC, and to identify miRNAs that control DTYMK expression levels within HCC cells.

## Materials And Methods

### Patient Samples

Tumor tissue and paracancerous samples were collected from 40 patients who had been clinically diagnosed with HCC at this hospital from January 2012 – July 2013 and who had undergone surgical tumor resection in the Department of Surgery at this hospital during this time period. Samples were paraffin-embedded and used for downstream histopathological and immunohistochemistry (IHC) examinations. Patient clinicopathological characteristics are compiled in **Table 1**.

**Table 1:** Clinicopathological characteristics of patient samples and expression of DTYMK in HCC.

Characteristics	No. of case (%)
**Age**	
<50	6 (15.0)
≥50	34 (85.0)
**Gender**	
Male	34 (85.0)
Female	6 (15.0)
**Liver cirrhosis**	
Yes	22 (55.0)
No	18 (45.0)
**AFP (ng/L)**	
<200	31 (77.5)
≥200	9 (22.5)
**ALT (U/L)**	
<60	29 (72.5)
≥60	11 (27.5)
**AST (U/L)**	
<40	29 (72.5)
≥40	11 (27.5)
**Tumor number**	
Single	23 (57.5)
Multiple	17 (42.5)
**Tumor size**	
<5cm	25 (62.5)
≥5cm	15 (37.5)
**Portal vein invasion**	
Yes	7 (17.5)
No	33 (82.5)
**TNM stage**	
I+II stage	35 (87.5)
III+IV stage	5 (12.5)

HCC, hepatocellular carcinoma; AFP, α-fetoprotein; ALT, alanine aminotransferase; AST, aspartate aminotransferase; TNM, tumor, node, metastasis.

### Cell Culture

The Huh7, SNU398, SK-HEP1, Hep3B, and HepG2 human HCC cell lines were purchased from the Chinese Academy of Sciences Cell Bank, and were grown in DMEM (Corning, VA, USA) containing 10% FBS (Gibco, CA, USA) in a 5% CO_2_ 37°C humidified incubator. Primary human hepatocytes (PHHs) were obtained by isolating samples of healthy liver tissue from patients undergoing liver resection and were cultured as in prior studies (17).

### Analyses of Public HCC Datasets

DTYMK expression in a large HCC patient cohort was assessed by querying The Cancer Genome Atlas liver HCC project (TCGA_LIHC), Gene Expression profiling interactive analysis (GEPIA), and UALCAN databases.

### RNA Extraction

RNAiso (TaKaRa, Otsu, Japan) was used to isolate total RNA from appropriate samples, after which a NanoDrop1000 instrument (Thermo Fisher, MA, USA) was used to quantify RNA levels in these samples. Following isolation, RNA was stored at −80°C.

### QRT-PCR

A PrimeScript™ RT Master Mix kit (TaKaRa, Otsu, Japan) was utilized to prepare cDNA based on provided instructions, while a miRcute Plus miRNA First-Strand cDNA Synthesis Kit (TIANGEN BIOTECH, China) was utilized when synthesizing cDNA for miRNA analyses. QRT-PCR reactions were conducted with a LightCycler^®^ 480 II (Roche, Switzerland) instrument and SYBR^®^ Premix Ex Taq™ II (TaKaRa, Otsu, Japan) based upon provided directions. U6 and GAPDH were used to normalize miRNA and mRNA relative expression, respectively, with the comparative Ct method being used for relative quantification. Primers used in this study are compiled in **Supplementary Table 1**.

### Cellular Transfection

GenePharma (Shanghai, China) synthesized miR-148b-3p mimics, inhibitors, and appropriate controls, as well as siRNAs specific for DTYMK. PrimeSTAR^®^ Max DNA Polymerase (TaKaRa, Otsu, Japan) was utilized to amplify a cDNA sequence corresponding to the DRYMK complementary determining sequence *via* PCR, after which this cDNA was cloned into the pcDNA3.1 vector (Invitrogen, CA, USA) using the BamHI and XhoI to yield the pcDNA3.1-DTYMK construct. This same amplification approach was also used to prepare cDNA sequences containing WT or mutant versions of the putative miR-148b-3p binding site, which were then cloned into the pGL6-miR vector (Beyotime, Shanghai, China) using the SacI and XhoI sites to yield pGL6-miR-DTYMK-wt and pGL6-miR-DTYMK-mut constructs, respectively. Lipofectamine 3000 (Invitrogen, CA, USA) was used to transfect cells with appropriate constructs based upon provided instructions. GeneChem (Shanghai, China) prepared the LV-shDTYMK lentiviral vector to suppress DTYMK expression. Cells were transduced with LV-shDTYMK or a control lentivirus (LV-control) together with 5 μg/mL Polybrene (Sigma-Aldrich, St. Louis, MO, USA), after which puromycin (1 μg/mL) was utilized to select for successfully transduced cells. All construct sequences discussed in this section are compiled in **Supplementary Table 1**.

### Immunohistochemistry (IHC)

Xylene was first used to deparaffinize tumor tissue sections, after which samples were rehydrated with ethanol, incubated with an antigen retrieval solution (Dako, Denmark) for 45 minutes, and treated for 10 minutes with 3% H_2_O_2_ prior to blocking for 20 minutes at room temperature (RT) using a protein block solution (Dako. Denmark). Sections were then incubated at 4°C with antibodies specific for DTYMK (ab154867, Abcam, 1:100 dilution, Cambridge, UK), MET, ERK2, IL-6, and NF-KB overnight, after which they were probed using appropriate secondary antibodies (Abcam, Cambridge, UK) for 30 minutes. An ABC reagent (Abcam, Cambridge, UK) was then applied to all samples for an additional 30 minutes, after which DAB (Dako, Denmark) was applied to detect protein staining. Sections were then counterstained with hematoxylin, mounted, and imaged with Ts2 and acquisition software (Nikon, Tokyo, Japan).

### Luciferase Reporter Assay

Putative interactions between miR-148b-3p and DTYMK were assessed through the use of a Dual-Luciferase Reporter Assay System (Promega, Madison, WI, USA) based on provided directions. Briefly, Hep3B cells were transfected with recombinant pGL6-miR-DTYMK or pGL6-miR-DTYMK-mut plasmids along with miR-148b-3p mimics or appropriate negative control (NC) constructs. At 48 h post-transfection, luciferase activity was then measured.

### Western Blotting

Appropriately treated cells were collected, rinsed with chilled PBS, and lysed using cold RIPA lysis buffer supplemented with 1% Protease Inhibitor Cocktail (Sigma-Aldrich, St. Louis, MO, USA). Next, protein samples were separated *via* 4-20% SDS-PAGE and transferred to PVDF membranes (Millipore, Billerica, MA, USA) that were subsequently blocked using 1% non-fat milk (Santa Cruz) for 1 h. Blots were then probed overnight with appropriate primary antibodies (anti-DTYMK or anti-GAPDH) at 4°C, followed by incubation with HRP Goat Anti-Rabbit IgG (H + L) (Abcam, Cambridge, UK). A Bio-Rad ChemiDoc XRS+ system was then used to detect protein bands, with GAPDH being used to normalize DTYMK protein levels.

### Cell Proliferation Assay

A Cell Counting Kit-8 (CCK8) assay kit from Dojindo Laboratories (Japan) was used to evaluate tumor cell proliferation. Briefly, we added 3000 cells/well of a 96-well plate, and after 0, 24, 48, or 72 h of culture we added CCK8 reagent to appropriate wells and incubated plates for an additional 2 h at 37°C. We then utilized a microplate reader (Bio-Rad, CA, USA) to quantify absorbance (OD) at 450 nm for each well, and plotted the resultant values to generate proliferation curves. In addition, EdU incorporation staining assays were conducted with an EdU Kit (Beyotime, Shanghai, China) based upon provided directions. ImageJ (NIH, USA) was used for all analyses of the resultant images.

### Wound Healing Assay

HCC cells were plated until confluent, at which time a sterile micropipette tip was used to generate a straight scratch wound in the cell monolayer at 24 h post-transfection. PBS was then used to wash away detached cells, after which the remaining cells were cultured for 24 in an appropriate culture medium, with wound area being assessed at 0 and 24 h post-wounding.

### Transwell Assays

Appropriately treated HCC cells were collected at 24 h post-transfection and were rinsed in PBS prior to addition to the top chamber of a Transwell insert that either had or had not been coated using Matrigel for invasion and migration assay, respectively. The lower chamber was then filled with DMEM containing 10% FBS, and cells were incubated for 12-18 h after which 4% paraformaldehyde was used to fix invasive cells. These cells were then stained with a 0.1% DAPI solution and were counted with the Ts2 and acquisition software (Nikon, Tokyo, Japan).

### *In Vivo* Studies

Briefly, athymic nude BALB/c mice (female; 6 weeks old) were subcutaneously implanted with 2 × 10^6^ HCC cells that had been transduced with LV-Control or LV-shDTYMK constructs. Tumor growth was then assessed every other day, and tumor volume (V) was calculated as follows: V = (W × W × L)/2, with W and L corresponding to tumor width and length, respectively. On day 24 post-implantation, mice were sacrificed and the number of metastatic nodules in the liver of each animal were quantified. In addition, pulmonary metastases were identified *via* hematoxylin and eosin (H&E) staining. The Institutional Ethics Committee of the Affiliated Hospital of Qingdao University approved all animal studies.

### dTTP Extraction and Quantification

Initially, extracts were prepared from 10^6^ cells by treating them using 60% ice-cold methanol, after which samples were immersed in 100°C dry bath for 3 minutes. Extracts were then dried under vacuum conditions as in prior studies (18). The resultant residue was then resuspended in 80 μl of nuclease-free dH_2_O, after which dTTP levels were quantified as in prior reports (19).

### Statistical Analysis

All data were analyzed using SPSS v18.0 (IBM, USA) and GraphPad Prism 6.0. Data were compared *via* unpaired or paired Student’s t-tests, and are given as means with standard deviations (SD). p < 0.05 was the significance threshold. *p < 0.05; **p < 0.01.

## Results

### HCC Tumors and Cell Lines Exhibit DTYMK Expression That Correlates With Poor Prognosis

**Figure 1:**
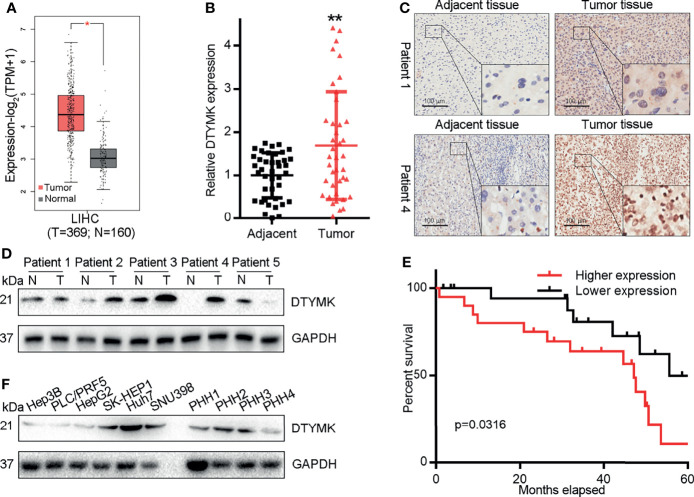
HCC is associated with DTYMK upregulation. **(A)** DTYMK levels in human HCC tumor and non-tumor tissues in the GEPIA database. **(B)** DTYMK expression was assessed *via* qRT-PCR in clinical HCC tumor and paracancerous tissues (n = 40). **(C)** DTYMK protein levels were assessed *via* IHC in HCC tumor and paracancerous tissues. Scale bar, 100 μm. **(D)** DTYMK protein levels were assessed *via* IHC in HCC tumor and paracancerous tissues *via* Western blotting. **(E)** The relationship between DTYMK mRNA levels and survival were assessed in 40 HCC patients *via* Kaplan–Meier analyses. **(F)** DTYMK protein levels were assessed *via* Western blotting in Hep3B, PLC/PRF5, HepG2, SK-HEP1, Huh7, and SNU398 cells and primary human hepatocytes (PHH-1, 2, 3 and 4). Data are means ± SD. *p < 0.05; **p < 0.01; Student’s t-tests and Kaplan-Meier analyses.

We began by assessing HCC patients in the GEPIA2 (http://gepia2.cancer-pku.cn/#index) (**Figure 1A**) and UALCAN (http://ualcan.path.uab.edu/) (**Supplementary Figure 1A**) databases. Analyses of these publically available datasets revealed that DTYMK expression levels were significantly higher in HCC tumor tissues relative to healthy tissue samples. We additionally confirmed these findings by assessing DTYMK mRNA expression in 40 pairs of HCC tumor and paracancerous tissue samples, which confirmed that DTYMK expression was increased 1.6-fold in HCC tumors relative to healthy tissues (**Figure 1B**). IHC staining further supported these findings, revealing markedly enhanced DTYMK protein staining in HCC samples (**Figure 1C**), with Western blotting additionally confirming the relative increase of DTYMK protein levels in tumors relative to noncancerous tissues (**Figure 1D**).

To investigate the frequency of DTYMK regulation in HCC, we examined the expression using qRT-PCR in 40 fresh-frozen HCC tissues, including 35 cases at clinical stage I and II, 5 cases at clinical stage III and IV (**Table 1**). Then we analyzed the association between DTYMK and the clinicopathological features of HCC. As shown in **Table 2**, strong associations were observed between DTYMK expression and AFP level (p = 0.008). However, the expression of DTYMK was not associated with age (p *=*0.077), gender (p = 0.077), liver cirrhosis (p = 1.000), ALT (p = 0.288), AST ( p = 0.723), tumor number ( p = 0.110), tumor size (p = 0.327), portal vein invasion (p = 0.677) or TNM stage (p = 0.446). Spearman analysis of correlation between DTYMK and clinicopathological features revealed that the expression of DTYMK was significantly correlated with AFP level (p = 0.007) (**Table 3**). Study of cox-regression was used to decide whether DTYMK would function as a risk factor. **Table 4** demonstrates Cox regression univariate analysis results that indicated how elevated DTYMK expression correlated with a substantially raised likelihood of death in HCC patients (p < 0.05). Results of the multivariate analysis: there was no statistically significant difference in DTYMK expression (p *=* 0.121) and AFP level (p = 0.112) (**Table 4**).

**Table 2:** Correlation between DTYMK expression and clinicopathologic characteristics of HCC patients.

Characteristics DTYMK	DTYMK Expression		
	Low or None, No. Cases	High, No. Cases	p Value
**Age**			
<50	1	5	0.077
≥50	19	15	
**Gender**			
Male	15	19	0.077
Female	5	1	
**Liver cirrhosis**			
Yes	9	9	1.000
No	11	11	
**AFP (ng/L)**			
<200	19	12	**0.008**
≥200	1	8	
**ALT (U/L)**			
<60	13	16	0.288
≥60	7	4	
**AST (U/L)**			
<40	15	14	0.723
≥40	5	6	
**Tumor number**			
Single	14	9	0.110
Multiple	6	11	
**Tumor size**			
<5cm	14	11	0.327
≥5cm	6	9	
**Portal vein invasion**			
Yes	3	4	0.677
No	17	16	
**TNM stage**			
I+II stage	18	17	0.446
III+IV stage	2	3	

P values were calculated using chi-square test. Bold numbers indicate significant differences (P < 0.05). HCC, hepatocellular carcinoma; AFP, α-fetoprotein; ALT, alanine aminotransferase; AST, aspartate aminotransferase; TNM, tumor, node, metastasis.

**Table 3:** Spearman analysis of correlation between DTYMK and clinicopathological.

Variables	DTYMK Expression Level	
	Spearman Correlation	p Value
Age (years, <50 vs. ≥50)	-0.280	-0.080
Gender (male/female)	-0.280	-0.080
Liver cirrhosis (yes/no)	0.000	1.000
AFP (ng/L, <200 vs. ≥200)	**0.419**	**0.007**
ALT (U/L, <60 vs. ≥60)	-0.168	0.300
AST (U/L, <40 vs. ≥40)	0.056	0.731
Tumor number (single/multiple)	0.253	0.115
Tumor size (cm, <5 vs. ≥5)	0.155	0.340
Portal vein invasion (yes/no)	0.066	0.687
TNM stage (I+II vs III+IV)	0.223	0.168

Bold numbers indicate significant differences (P < 0.05). HCC, hepatocellular carcinoma; AFP, α-fetoprotein; ALT, alanine aminotransferase; AST, aspartate aminotransferase; TNM, tumor, node, metastasis.

**Table 4:** Univariate and multivariate analyses of various prognostic parameters in patients with HCC Cox-regression analysis.

	Univariate Analysis			Multivariate aAnalysis		
	p Value	Hazard Ratio	95% Confidence Interval	p Value	Hazard Ratio	95% Confidence Interval
**DTYMK**	**0.039**	2.593	1.052-6.392	0.121	2.14	0.818-5.598
**AFP (ng/L, <200 vs. ≥200)**	**0.028**	3.174	1.132-8.899	0.112	2.433	0.813-7.218

Bold numbers indicate significant differences (P < 0.05). HCC, hepatocellular carcinoma; AFP, α-fetoprotein.

Through Kaplan-Meier survival analyses, we additionally determined that elevated DTYMK expression was associated with decreased overall survival (OS) in HCC patients (p = 0.0316; **Figure 1E**), and this finding was supported by prognostic assessments of the GEPIA2 and UALCAN datasets which confirmed that elevated DTYMK expression was associated with poorer OS (**Supplementary Figures 1B, C**). Together, these data provide evidence that DTYMK is a prognostic factor in HCC. When we additionally assessed the expression of this gene in PHHs and in the Hep3B, PLC/PRF5, HepG2, SK-HEP1, Huh7, and SNU398 liver cancer cell lines, we found that DTYMK was similarly overexpressed in tumor cells relative to levels in healthy PHHs (**Figure 1F**). This thus suggests that DTYMK may play an oncogenic role in HCC such that its elevated expression is correlated with a poorer HCC patient prognosis.

### DTYMK Knockdown Suppresses Tumor Growth *In Vitro* and *In Vivo*

**Figure 2:**
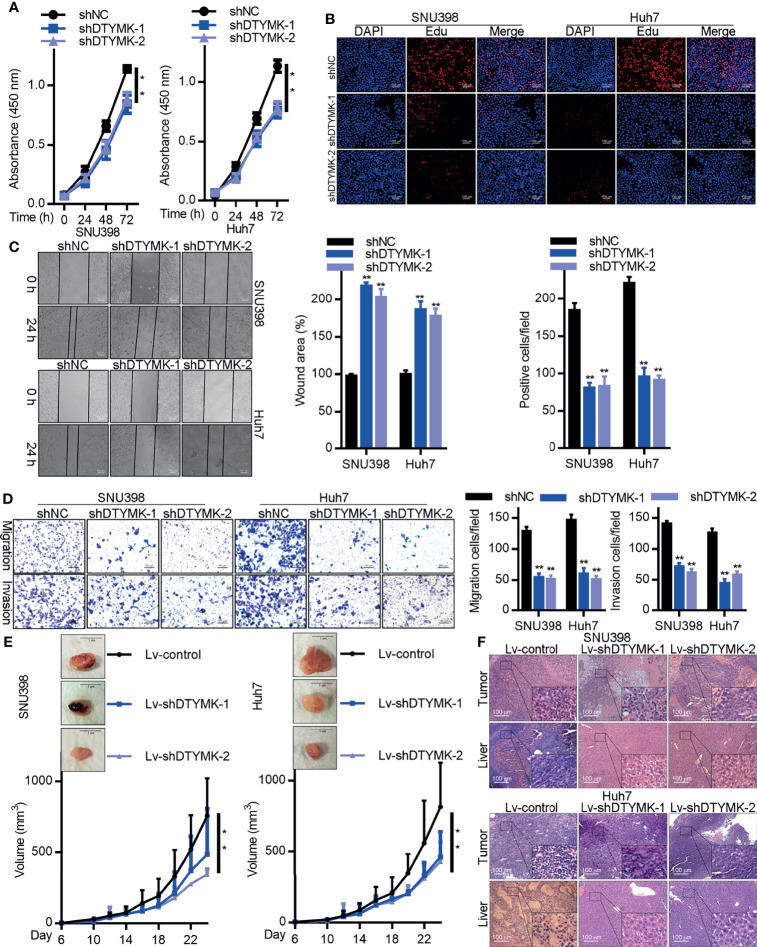
DTYMK promotes HCC tumorigenesis. **(A)** The proliferation of SNU398 (left) and Huh7 (right) cells in which DTYMK was knocked down was assessed *via* CCK-8 assay and **(B)** EdU incorporation staining assay. Scale bars 100 μm. **(C)** A wound healing assay was used to assess the migration of cells treated as in **(A)**. Scale bar, 500 μm. **(D)** The migratory and invasive activity of cells treated as in **(A)** was assessed *via* Transwell assay. Scale bar, 100 μm. **(E)** The impact of DTYMK knockdown on the growth of SNU398 (left) and Huh7 (right) tumors. Tumor volume was measured every other day, and animals were euthanized 24 days post-implantation, at which time tumors were excised. n=6 mice/group. Scale bar, 1 cm. **(F)** H&E staining analysis of the tumor and liver section in mice xenografts. Scale bars, 100 μm. Data are means ± SD. *p < 0.05; **p < 0.01; Student’s t-tests.

The biological function of DTYMK in HCC was assessed by employing an RNAi approach, with both the shDTYMK-1 and shDTYMK-2 constructs achieving significant inhibition of the expression of this gene (**Supplementary Figure 1D**). A CCK8 kit was then used to assess proliferation following DTYMK knockdown (**Figure 2A**), with an EdU incorporation staining approach further being employed to quantify the proliferation of SNU398 and Huh7 cells following the knockdown of this oncogene (**Figure 2B**). These analyses both confirmed that suppressing the expression of DTYMK markedly suppressed HCC tumor cell proliferation. Wound healing assays (**Figure 2C**) and Transwell migration and invasion assays further revealed that DTYMK knockdown impaired the migratory and metastatic activity of these HCC cell lines, with a roughly 2-fold reduction in the number of invasive DTYMK-knockdown cells being observed relative to control cells in Transwell assays (**Figure 2D**).

We next stably transduced Huh7 and SNU398 cells with Lv-shDTYMK-1 and Lv-shDTYMK-2 constructs in order to stably knock down DTYMK expression there (**Supplementary Figure 1E**). These cells or appropriate controls were then subcutaneously implanted into BALB/c nude mice, and tumor growth was monitored over time, revealing that suppressing DTYMK expression markedly inhibited the ability of these HCC tumors to grow *in vivo* (**Figure 2E**). H&E staining further revealed that fewer and smaller liver metastases were evident in mice that had been implanted with DTYMK-knockdown tumors relative to mice transplanted with control tumors (**Figure 2F**). We similarly overexpressed DTYMK in Hep3B cells, and determined that this increased their malignancy (**Supplementary Figure 2**). Overall, these findings indicated that DTYMK functions as an important oncogene in HCC, and suppressing its expression can impair the growth and metastasis of HCC *in vitro* and *in vivo.*

### MiR-148b-3p Targets DTYMK in Liver Cancer Cells

**Figure 3:**
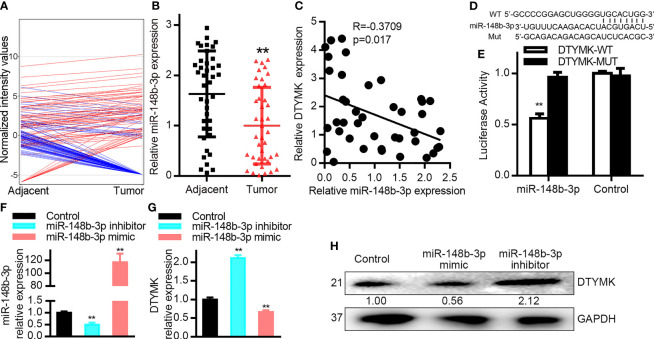
MiR-148b-3p targets DTYMK in HCC cells. **(A)** miRNA expression profiles were compared in tumor and paracancerous tissues *via* Agilent Human miRNA microarray. **(B)** MiR-148b-3p expression was assessed *via* qRT-PCR in HCC tumor and paracancerous tissues (n = 40). **(C)** Correlations between the expression of DTYMK and miR-148b-3p in HCC patient samples (n = 40 samples). p *=* 0.017, R = −0.3709; Spearman correlation analysis. **(D)** Schematic of the putative miR-148b-3p binding site in the DTYMK 3’-UTR. WT or MUT DTYMK 3’-UTR constructs were cloned into the pGL6-miR luciferase reporter vector. **(E)** Co-transfection of Hep3B cells with miR-148b-3p and the WT DTYMK 3’- UTR vector was associated with reduced luciferase activity, whereas the activity of the MUT vector was not affected. As a control, Hep3B cells were transfected with the luciferase reporters and a negative control miRNA, or with vectors alone. **(F)** MiR-148b-3p expression levels in Hep3B cells transfected with a miR-148b-3p inhibitor or mimic. **(G, H)** DTYMK mRNA and protein levels in cells treated as in **(F)**. Data are means ± SD. **p < 0.01; Student’s t-tests and Spearman’s correlation analyses.

We next sought to determine what factors were responsible for the endogenous regulation of DTYMK in HCC cells by comparing the expression levels of different miRNAs between tumor and paracancerous tissues in an HCC patient that exhibited robust intratumoral DTYMK expression (Patient 4; **Supplementary Figure 3A**). We observed clear differences in miRNA expression patterns between these two tissues, with 107 and 66 miRNAs ultimately being found to be up- and downregulated, respectively, in tumor tissues relative to paracancerous tissues (**Figure 3A** and **Table 5**).

**Table 5:** Dysregulation of miRNAs in hepatocellular carcinoma tissue.

miRNA Name	Score (d)
Decreased expression (107)	
hsa-miR-130b-3p	-201.53
hsa-miR-551b-3p	-105.02
hsa-miR-532-5p	-83.179
hsa-miR-4465	-80.661
hsa-miR-224-5p	-48.17
hsa-miR-374b-5p	-46.651
hsa-miR-4721	-44.935
hsa-miR-96-5p	-44.878
hsa-miR-152	-44.005
hsa-miR-1246	-42.518
hsa-miR-301a-3p	-41.643
hsa-miR-362-5p	-40.382
hsa-miR-216a-5p	-36.504
hsa-miR-642b-3p	-36.502
hsa-miR-4442	-35.877
hsa-miR-3198	-34.515
hsa-miR-4713-3p	-34.383
hsa-miR-6130	-32.391
hsa-miR-532-3p	-31.683
hsa-miR-34b-5p	-30.685
hsa-miR-1181	-30.044
hsa-miR-4787-3p	-30.036
hsa-miR-363-3p	-29.796
hsa-miR-6124	-29.672
hsa-miR-362-3p	-29.644
hsa-miR-4669	-28.963
hsa-miR-1234-3p	-28.726
hsa-miR-6508-5p	-28.621
hsa-miR-1825	-28.496
hsa-miR-1229-5p	-28.085
hsa-miR-6131	-27.95
hsa-miR-4515	-27.769
hsa-miR-1304-3p	-26.832
hsa-miR-142-5p	-26.013
hsa-miR-502-3p	-25.156
hsa-miR-149-5p	-25.084
hsa-miR-205-3p	-25.049
hsa-miR-766-3p	-24.784
hsa-miR-224-3p	-24.371
hsa-miR-148b-3p	-23.375
hsa-miR-4428	-22.503
hsa-miR-18a-5p	-21.047
hsa-miR-1973	-20.209
hsa-miR-4728-5p	-20.196
hsa-miR-98-5p	-18.619
hsa-miR-361-3p	-18.277
hsa-miR-500a-3p	-17.953
hsa-miR-9-3p	-17.065
hsa-miR-183-5p	-16.886
hsa-miR-4306	-16.605
hsa-miR-129-2-3p	-15.769
hsa-miR-125a-3p	-15.731
hsa-miR-4651	-14.238
hsa-miR-181b-5p	-13.926
hsa-miR-95	-13.868
hsa-miR-222-3p	-13.832
hsa-miR-4430	-13.829
hsa-miR-4746-3p	-13.713
hsa-miR-663a	-13.256
hsa-miR-129-1-3p	-13.254
hsa-miR-194-3p	-13.186
hsa-miR-4656	-12.948
hsa-miR-4484	-12.936
hsa-miR-4745-5p	-12.887
hsa-miR-4323	-12.879
hsa-miR-4769-3p	-12.822
hsa-miR-3692-5p	-12.801
hsa-miR-3676-3p	-12.801
hsa-miR-3127-5p	-12.725
hsa-miR-371a-5p	-12.641
hsa-miR-1587	-12.527
hsa-miR-1914-3p	-12.507
hsa-miR-484	-12.118
hsa-miR-652-3p	-12.112
hsa-miR-454-3p	-12.039
hsa-miR-29c-5p	-11.885
hsa-miR-186-5p	-11.868
hsa-miR-4749-3p	-11.747
hsa-miR-188-5p	-11.74
hsa-miR-885-3p	-11.718
hsa-miR-4271	-11.626
hsa-miR-204-5p	-11.623
hsa-miR-143-3p	-11.366
hsa-miR-425-3p	-11.348
hsa-let-7f-1-3p	-10.728
hsa-miR-18b-5p	-10.378
hsa-miR-4652-3p	-8.4989
hsa-miR-6509-5p	-7.7097
hsa-miR-501-3p	-7.1944
hsa-miR-505-5p	-6.8829
hsa-miR-216b	-6.3181
hsa-miR-135a-3p	-5.8261
hsa-miR-15b-5p	-4.9773
hsa-miR-155-5p	-4.7231
hsa-miR-425-5p	-3.6448
hsa-miR-93-5p	-3.5386
hsa-miR-106b-5p	-3.2748
hsa-miR-660-5p	-3.1927
hsa-miR-642a-3p	-2.7251
hsa-miR-21-3p	-2.5885
hsa-miR-185-5p	-2.5607
hsa-miR-324-5p	-2.4888
hsa-miR-4530	-2.4094
hsa-miR-25-3p	-2.1823
hsa-miR-331-3p	-2.1419
hsa-miR-574-5p	-2.0548
hsa-miR-423-5p	-2.0068
Increased expression (66)	
hsa-miR-6088	2.03899
hsa-miR-30e-3p	2.04208
hsa-miR-20a-5p	2.07775
hsa-miR-4286	2.10388
hsa-miR-1238-3p	2.10871
hsa-miR-5787	2.15169
hsa-miR-22-3p	2.1741
hsa-let-7b-3p	2.18946
hsa-miR-320b	2.1972
hsa-miR-181a-5p	2.20178
hsa-miR-638	2.2344
hsa-miR-320c	2.2404
hsa-miR-664b-3p	2.26651
hsa-miR-320e	2.35334
hsa-miR-2861	2.39617
hsa-miR-4459	2.40427
hsa-miR-92a-3p	2.41235
hsa-miR-3656	2.44972
hsa-miR-100-5p	2.45715
hsa-miR-29c-3p	2.45883
hsa-miR-365a-3p	2.45948
hsa-miR-146a-5p	2.50713
hsa-miR-122-3p	2.52101
hsa-miR-1260b	2.533
hsa-miR-1225-5p	2.5624
hsa-miR-483-3p	2.57767
hsa-miR-4281	2.58026
hsa-miR-29a-3p	2.59002
hsa-miR-27b-3p	2.61436
hsa-miR-320d	2.64953
hsa-miR-193a-3p	2.71566
hsa-miR-150-5p	2.74278
hsa-miR-23b-3p	2.74794
hsa-miR-1260a	2.88221
hsa-miR-122-5p	2.88527
hsa-miR-193b-3p	2.90692
hsa-miR-125b-5p	2.92258
hsa-miR-27a-3p	2.94485
hsa-miR-23a-3p	3.22285
hsa-miR-378i	3.37698
hsa-miR-378a-3p	3.54521
hsa-miR-101-3p	3.63335
hsa-miR-223-3p	4.0117
hsa-miR-4741	4.03577
hsa-miR-125a-5p	4.05035
hsa-let-7e-5p	4.24897
hsa-miR-6068	4.89593
hsa-miR-130a-3p	5.47268
hsa-miR-145-5p	6.15763
hsa-miR-451a	7.05553
hsa-miR-455-3p	7.21078
hsa-miR-424-5p	7.83366
hsa-miR-200b-3p	8.12075
hsa-miR-10a-5p	11.4564
hsa-miR-4270	16.2961
hsa-miR-199a-3p	40.4312
hsa-miR-483-5p	54.4347
hsa-miR-338-3p	55.4635
hsa-miR-486-5p	57.0137
hsa-miR-378a-5p	66.5858
hsa-miR-455-5p	71.8033
hsa-miR-200a-3p	87.4733
hsa-miR-193a-5p	98.062
hsa-miR-375	121.056
hsa-miR-214-3p	245.259
hsa-miR-199a-5p	677.445

Of the 66 significantly downregulated miRNAs identified in this analyses, we next focused on assessing miR-148b-3p. We then assessed the expression of miR-148b-3p in all 40 pairs of HCC tumors and paracancerous tissues *via* qRT-PCR (**Figure 3B**), confirming that this miRNA is downregulated in HCC tumors. Similar findings were also observed in HCC cell lines, with significant miR-148b-3p downregulation in Huh7, SNU398, SK-HEP1, Hep3B, and HepG2 cells relative to PHHs (1–4) (**Supplementary Figure 3B**).

We additionally found that miR-148b-3p was significantly downregulated in HCC patient samples (**Figure 3B**). Importantly, in our 40 pairs of HCC tumor and paracancerous tissues, we detected a negative correlation between miR-148b-3p and DTYMK mRNA expression levels (**Figure 3C**). The predictive TargetScan tool additionally identified miR-148b-3p as a putative regulator of DTYMK capable of binding to a conserved region in the 3’-UTR of the DTYMK mRNA (**Figure 3D**). We next constructed luciferase reporter vectors containing WT or mutant (mut) versions of this putative 3’-UTR binding sequence (**Figure 3D**), and conducted a luciferase reporter assay using Hep3B cells. This analysis revealed that miR-148b-3p can directly target the WT but not the mutant version of the DTYMK 3’-UTR (**Figure 3E**), confirming the regulatory relationship between this miRNA-mRNA pair. In order to understand the degree to which miR-148b-3p regulates DTYMK expression levels in HCC cells, we next transfected these cells with miR-148b-3p mimic or inhibitor constructs (**Figure 3F**). Cells transfected with miR-148b-3p inhibitors exhibited a 1.5-fold increase in DTYMK protein and mRNA levels, whereas these levels were reduced in cells transfected with miR-148b-3p mimics (**Figures 3G, H**). In contrast, altering the expression of DTYMK did not affect miR-148b-3p expression levels in these HCC cells. These data thus suggest that miR-148b-3p is an upstream regulator of DTYMK in HCC cells, suppressing its expression.

### MiR-148b-3p Targets DTYMK to Suppress Oncogenic Activity in HCC Cells

**Figure 4:**
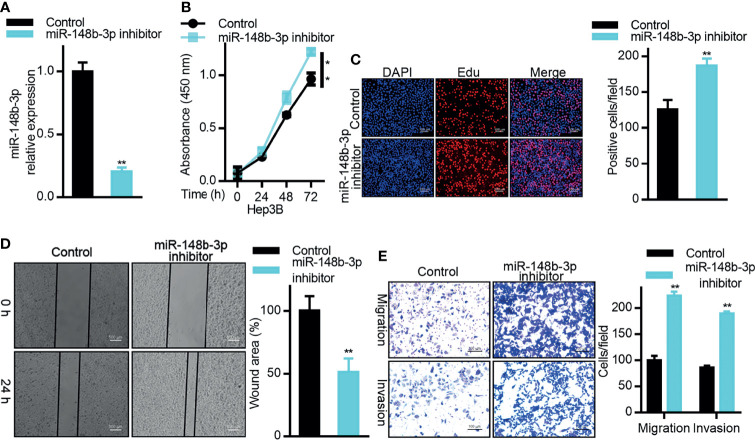
MiR-148b-3p functions as a tumor suppressor in HCC. **(A)** miR-148b-3p expression was assessed in Hep3B cells transfected with a miR-148b-3p inhibitor. **(B)** The proliferation of cells treated as in **(A)** was assessed *via* CCK-8 assay and *via* **(C)** EdU incorporation staining assay. Scale bar, 100 μm. **(D)** A wound healing assay was used to assess the migration of cells treated as in **(A)**. Scale bar, 500 μm. **(E)** The migratory and invasive activity of cells treated as in **(A)** was assessed *via* Transwell assay. Scale bar, 100 μm. Data are means ± SD. *p < 0.05; **p < 0.01; Student’s t-tests.

As miR-148b-3p suppresses the expression of DTYMK in HCC cells, this indicates that this miRNA may function as a tumor suppressor. To test this possibility, we therefore evaluated the impact of miR-148b-3p inhibition on HCC cells (**Figure 4A**). CCK8 and EdU assays confirmed that inhibition of this miRNA enhanced Hep3B cell proliferation (**Figures 4B, C**), while also enhancing the metastatic and invasive activities of these cells in Transwell and wound healing assays (**Figures 4D, E**).

**Figure 5:**
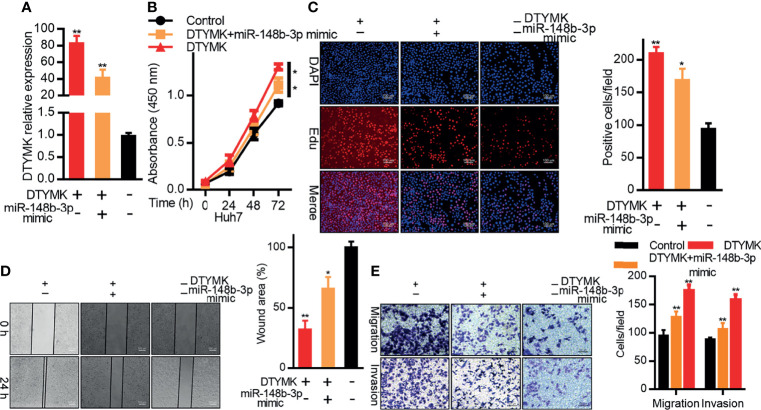
MiR-148b-3p suppresses the pro-tumorigenic activity of DTYMK. **(A)** DTYMK mRNA expression was assessed in Hep3B cells overexpressing DTYMK and co-transfected with a miR-148b-3p mimic. **(B)** The proliferation of cells treated as in **(A)** was assessed *via* CCK-8 assay and *via* **(C)** EdU incorporation staining assay. Scale bar, 100 μm. **(D)** A wound healing assay was used to assess the migration of cells treated as in **(A)**. Scale bar, 500 μm. **(E)** The migratory and invasive activity of cells treated as in **(A)** was assessed *via* Transwell assay. Scale bar, 100 μm. Data are means ± SD. *p < 0.05; **p < 0.01; Student’s t-tests.

We then explored the relationship between miR-148b-3p and DTYMK expression in Huh7 cells by co-transfecting them with miR-148b-3p mimics and DTYMK overexpression vectors. As expected, miR-148b-3p mimic transfection suppressed DTYMK expression at the mRNA level (**Figure 5A**). In subsequent CCK8 and EdU assays, we found that Huh7 cells co-transfected with DTYMK and miR-148b-3p were less proliferative than were cells overexpressing DTYMK alone (**Figures 5B, C**). miR-148b-3p mimic transfection also partially suppressed the migratory and invasive activity of cells overexpressing DTYMK (**Figures 5D, E**). Together, these findings indicate that miR-148b-3p functions as a tumor suppressor in HCC that can suppress tumor cell proliferation and migration at least in part by inhibiting the expression of DTYMK.

### MiRNA-148b-3p Modulated DTYMK-Associated dTTP Pyrimidine Biosynthesis

**Figure 6:**
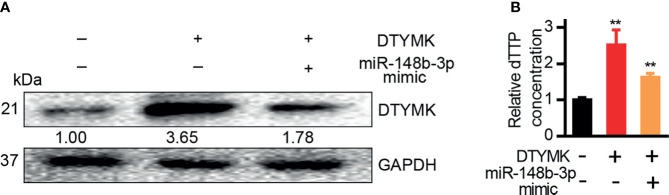
MiR-148b-3p suppression of DTYMK expression regulates intracellular dTTP levels. **(A)** DTYMK protein levels in cells transfected with miRNA-148b-3p mimics or/and DTYMK overexpression constructs. **(B)** Relative dTTP levels in cells transfected with miRNA-148b-3p mimic or/and DTYMK overexpression vectors **p < 0.01; Student’s t-tests.

DTYMK is a key enzyme necessary for dTTP biosynthesis in the context of pyrimidine metabolism (5), and both DTYMK expression and dTTP concentrations are elevated in the context of poorly differentiated HCC (5). We similarly found that DTYMK overexpression in Hep3B cells was associated with increased intracellular dTTP levels (**Figures 6A, B**), whereas miR-148b-3p overexpression in cells overexpressing DTYMK was associated with a reduction in these dTTP levels (**Figure 6B**).

## Discussion

Abundant research to date has highlighted the complex roles played by miRNAs in a range of diseases and physiological processes (20–22), but few studies have examined the association between miRNAs and DTYMK expression. Herein we demonstrated that DTYMK was able to enhance the proliferative and migratory activity of HCC cells, in line with prior evidence that this gene can promote HCC progression. We further characterized miR-148b-3p as a novel suppressor of DTYMK expression in HCC. Overexpressing miRNA-148b-3p can suppress the ability of DTYMK to enhance HCC cell proliferation and metastasis, and DTYMK expression was negatively correlated with that of miR-148b-3p in HCC patient samples. As such, these data suggest that miR-148b-3p may be a viable tool for the suppression of the DTYMK pathway in HCC cells.

Diverse miRNA-related regulatory activities have been identified and studies to date. In addition to their traditional roles as suppressors of post-transcriptional mRNA expression, Dragomir et al. have identified seven other putative miRNA functional roles including peptide coding, interacting with non-AGO proteins, targeting mitochondrial transcripts, activating transcription, targeting nuclear non-coding RNAs, upregulating protein expression, and activating Toll-like receptors (23). We did not observe any feedback inhibition of miR-148b-3p expression in response to DTYMK upregulation in the present analyses, suggesting that this miR-148b-3p/DTYMK axis is a unidirectional regulator of HCC cell proliferative dynamics.

Multiple recent studies have highlighted diverse miR-148b-3p target genes in a range of human tumor types (24–28), with some prior evidence demonstrating that this miRNA can directly target oncogenes including DNMT3b (25), CCK2R (26), DNMT1 (28), CEA (29), and AMPKα1 (30) in various cancers, thereby functioning as a tumor suppressor. Downregulation of miR-148b-3p cell proliferation, progression, and invasion in gastric cancer, pancreatic cancer, and NSCLC (27–30), while also being linked to poor prognosis and distant tumor metastasis in patients with breast and pancreatic cancer (30). Our results similarly suggest that the overexpression of miR-148b-3p reduces HCC cell growth and metastasis at least in part *via* suppressing DTYMK expression. However, whether this same regulatory relationship extends to other human tumor types remains to be clarified.

In summary, the results of this analysis underscore the fact that DTYMK is upregulated in HCC and plays a key role in the progression of this cancer type. We additionally identified miR-148b-3p as a novel endogenous regulator of DTYMK that also controls DTYMK-mediated dTTP production in HCC tumor cells. These findings suggest that this miR-148b-3p/DTYMK axis may thus be amenable to therapeutic targeting in patients with HCC.

## Data Availability Statement

The datasets presented in this study can be found in online repositories. The names of the repository/repositories and accession number(s) can be found in the article/**Supplementary Material**.

## Ethics Statement

The studies involving human participants were reviewed and approved by The Ethics Committee at the Affiliated Hospital of Qingdao University. The patients/participants provided their written informed consent to participate in this study. The animal study was reviewed and approved by The Ethics Committee at the Affiliated Hospital of Qingdao University.

## Author Contributions

SL designed the research and contributed majorly towards the writing of the manuscript. JQ and BT collected the tumors and tissues with their clinical information. CL and DC edited the manuscript and provided critical comments. GH were the major contributors in conducting the experiments, interpreting the data and drafting the manuscript. All authors read and approved the final manuscript and agree to be accountable for all aspects of the work in ensuring that questions related to the accuracy or integrity of any part of the work are appropriately investigated and resolved.

## Funding

This work was supported by the Natural Science Foundations of Shandong Province (ZR2021MH022).

## Conflict of Interest

The authors declare that the research was conducted in the absence of any commercial or financial relationships that could be construed as a potential conflict of interest.

## Publisher’s Note

All claims expressed in this article are solely those of the authors and do not necessarily represent those of their affiliated organizations, or those of the publisher, the editors and the reviewers. Any product that may be evaluated in this article, or claim that may be made by its manufacturer, is not guaranteed or endorsed by the publisher.

## Supplementary Material

Supplementary Figure 1 The prognostic relevance of DTYMK in HCC patients. **(A)** DTYMK expression levels were assessed in HCC patients in the UALCAN database with a p-value threshold of 0.05. **(B, C)** HCC patient OS was assessed for patients with low or high DTYMK expression (blue and red, respectively) in the GEPIA2 **(B)** and UALCAN **(C)** databases with a p-value threshold of 0.05. **(D)** DTYMK mRNA expression was assessed in SNU398 (left) and Huh7 (right) cells following the knockdown of this gene. **(E)** DTYMK mRNA expression was assessed in SNU398 (left) and Huh7 (right) cells following the lentiviral knockdown of this gene. Data are means ± SD. ***p < 0.05; ****p < 0.01; Student’s t-tests and Kaplan-Meier analyses.

Supplementary Figure 2 Overexpression of DTYMK promotes HCC tumorigenesis. **(A)** DTYMK protein and mRNA levels were assessed in Hep3B cells following DTYMK overexpression. **(B)** The proliferation of Hep3B cells overexpressing DTYMK was assessed *via* CCK8 assay, and **(C)** *via* EdU incorporation staining assay. Scale bar, 100 µm. **(D)** Wound healing assays were used to evaluate the migration of cells overexpressing DTYMK. Scale bar, 100 µm. **(E)** Transwell assays were used to measure the migration and invasion of Hep3B cells overexpressing DTYMK. Scale bar, 100 µm. Data are means ± SD. ***p < 0.05; ****p < 0.01; Student’s t-tests.

Supplementary Figure 3 Validation of MET, ERK2, IL-6, and NF-kB expression in HCC patients. **(A)** Levels of MET, ERK2, IL-6, and NF-kB were assessed *via* IHC. Scale bar, 100 µm. **(B)** QRT-PCR was assessed to assess miR-148b-3p expression in Huh7, SNU398, SK-HEP1, Hep3B, HepG2, PHH1, PHH2, PHH3, and PHH4 cells.
