# Supplementary figures and images for: MiR-148b-3p Regulates the Expression of DTYMK to Drive Hepatocellular Carcinoma Cell Proliferation and Metastasis
Source: Front Oncol. 2021 Dec 24;11:625566. doi: 10.3389/fonc.2021.625566 (PMC8739515; doi:10.3389/fonc.2021.625566)

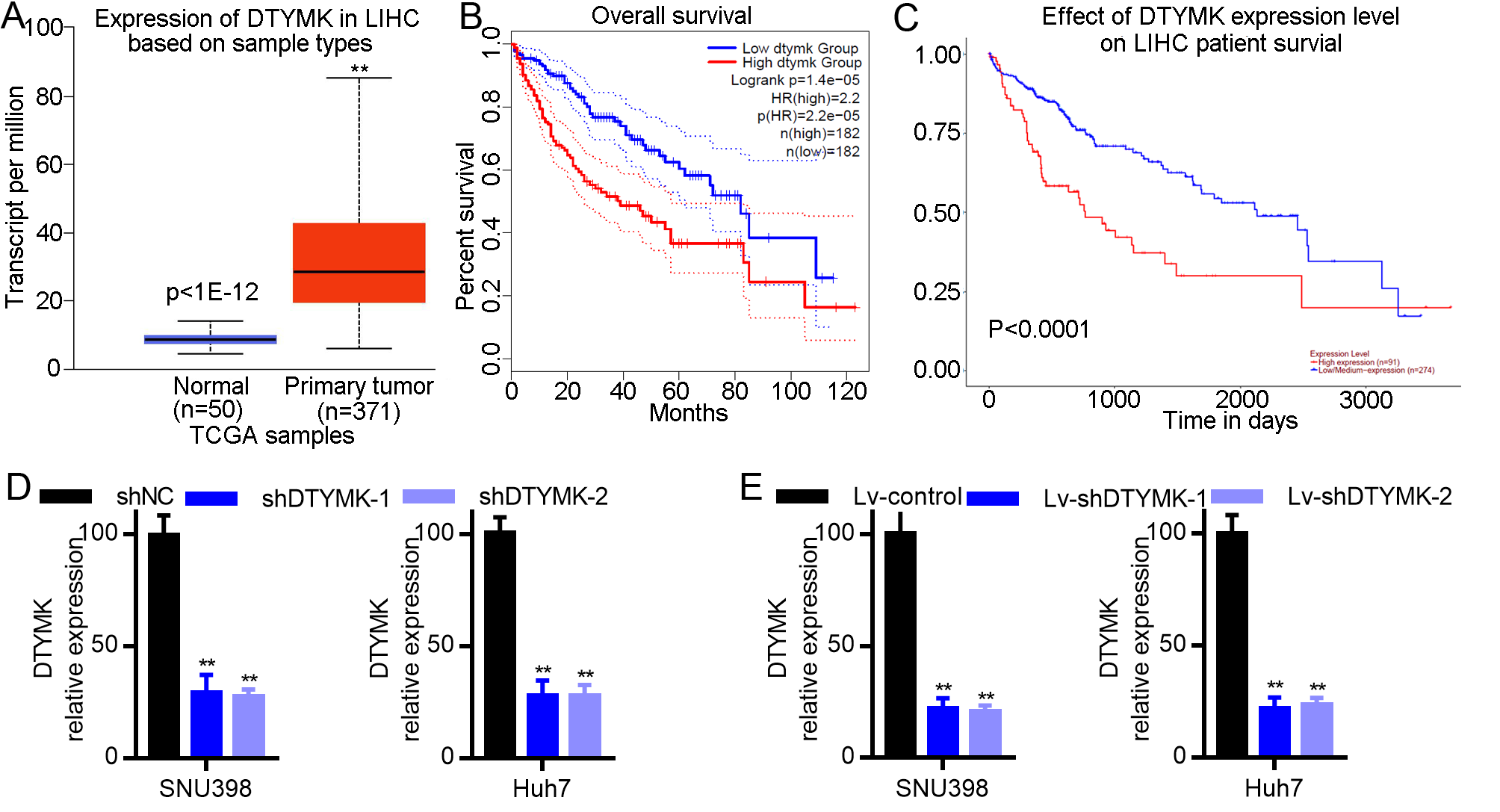

Supplement: Supplementary Figure 1 — The prognostic relevance of DTYMK in HCC patients. (A) DTYMK expression levels were assessed in HCC patients in the UALCAN database with a p-value threshold of 0.05. (B, C) HCC patient OS was assessed for patients with low or high DTYMK expression (blue and red, respectively) in the GEPIA2 (B) and UALCAN (C) databases with a p-value threshold of 0.05. (D) DTYMK mRNA expression was assessed in SNU398 (left) and Huh7 (right) cells following the knockdown of this gene. (E) DTYMK mRNA expression was assessed in SNU398 (left) and Huh7 (right) cells following the lentiviral knockdown of this gene. Data are means ± SD. *p < 0.05; **p < 0.01; Student’s t-tests and Kaplan-Meier analyses. [file Image_1.tif]

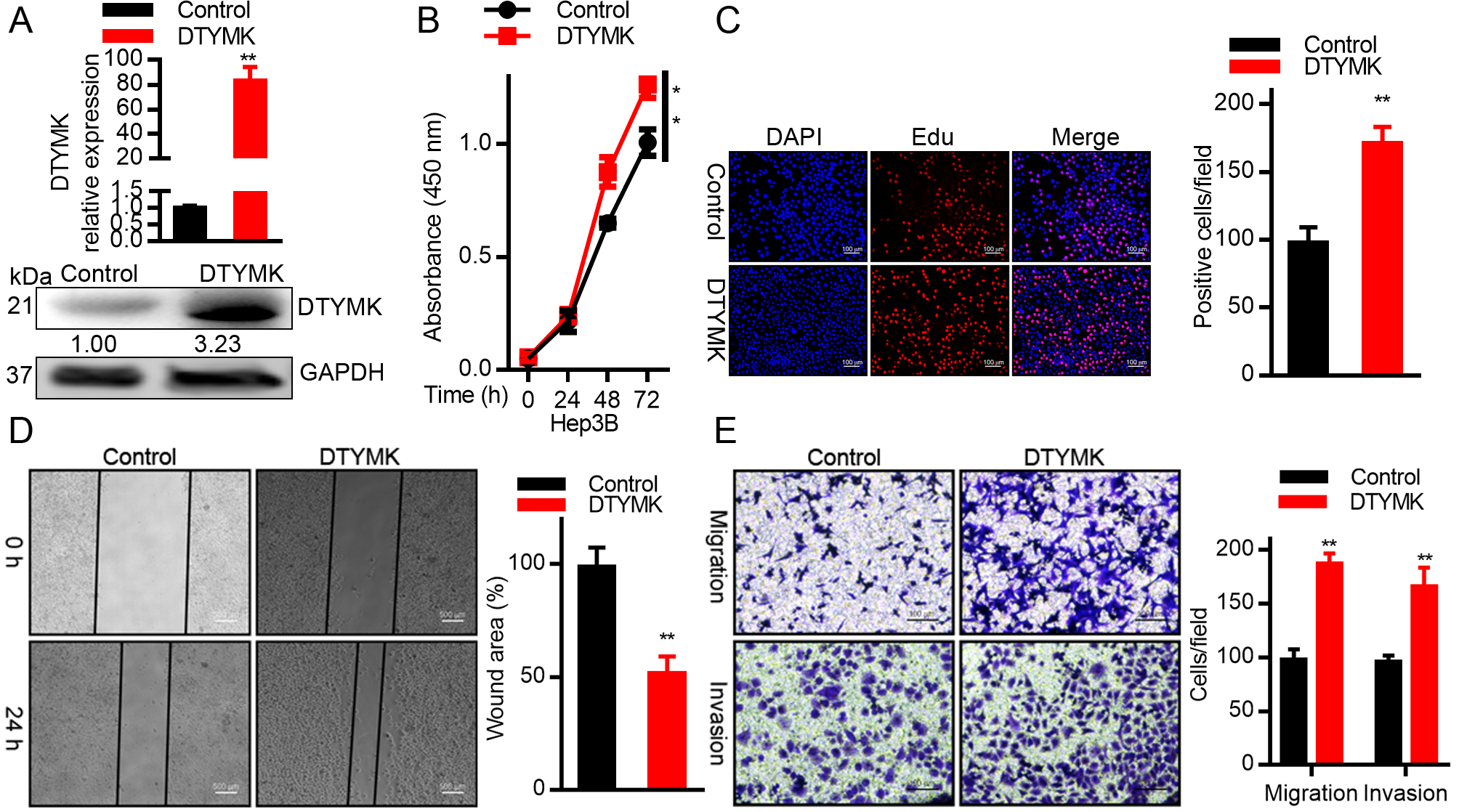

Supplement: Supplementary Figure 2 — Overexpression of DTYMK promotes HCC tumorigenesis. (A) DTYMK protein and mRNA levels were assessed in Hep3B cells following DTYMK overexpression. (B) The proliferation of Hep3B cells overexpressing DTYMK was assessed via CCK8 assay, and (C) via EdU incorporation staining assay. Scale bar, 100 µm. (D) Wound healing assays were used to evaluate the migration of cells overexpressing DTYMK. Scale bar, 100 µm. (E) Transwell assays were used to measure the migration and invasion of Hep3B cells overexpressing DTYMK. Scale bar, 100 µm. Data are means ± SD. *p < 0.05; **p < 0.01; Student’s t-tests. [file Image_2.tif]

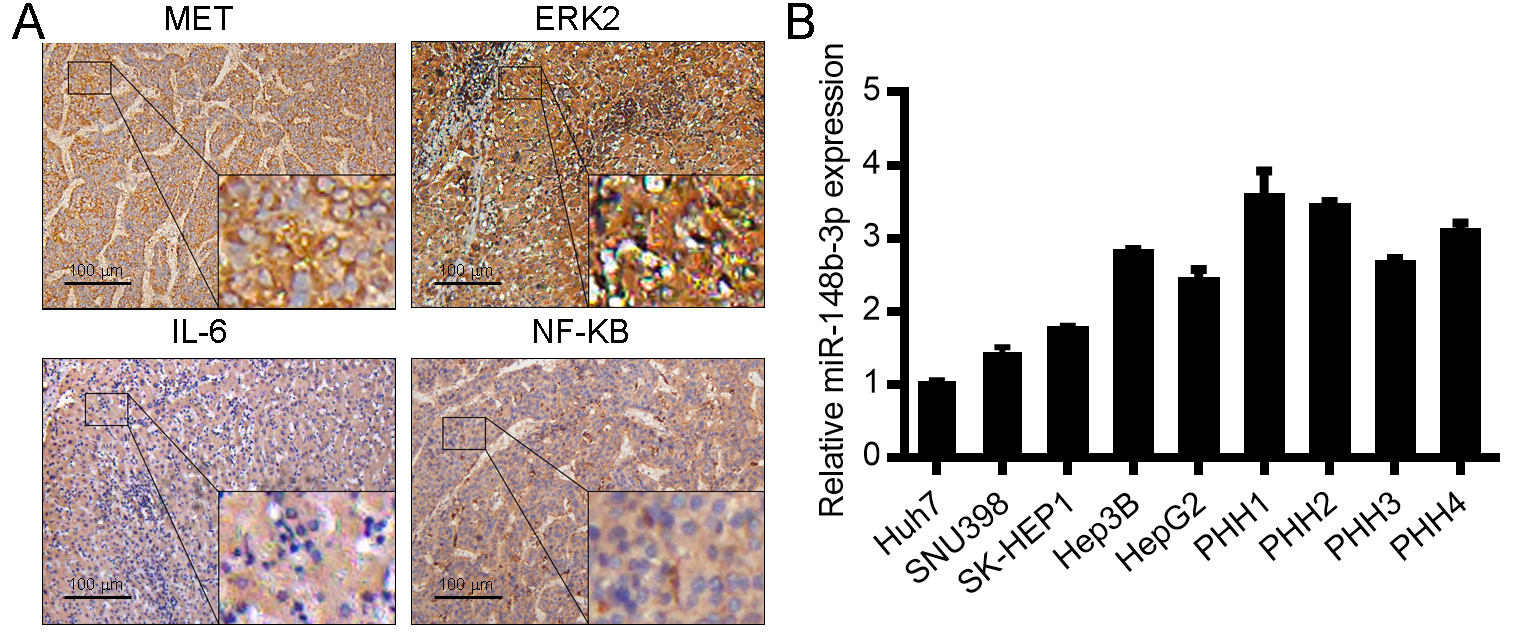

Supplement: Supplementary Figure 3 — Validation of MET, ERK2, IL-6, and NF-kB expression in HCC patients. (A) Levels of MET, ERK2, IL-6, and NF-kB were assessed via IHC. Scale bar, 100 µm. (B) QRT-PCR was assessed to assess miR-148b-3p expression in Huh7, SNU398, SK-HEP1, Hep3B, HepG2, PHH1, PHH2, PHH3, and PHH4 cells. [file Image_3.tif]
