# Supplementary material for: MiR-148b-3p Regulates the Expression of DTYMK to Drive Hepatocellular Carcinoma Cell Proliferation and Metastasis
Source: Front Oncol. 2021 Dec 24;11:625566. doi: 10.3389/fonc.2021.625566 (PMC8739515; doi:10.3389/fonc.2021.625566)
Supplement: Supplementary file 4 [file Table_1.docx]

Supplementary Table 1: Sequences of primers for qRT-PCR, vector construction

| **Primers for qRT-PCR** | Sequences (5′-3′) | |  |
| --- | --- | --- | --- |
| DTYMK (Forward) | | TGCAAATCGCTGGGAACAAGT | |
| DTYMK (Reverse) | | GCGTATCTGTCCACGACGAG | |
| GAPDH (Forward) | | GTCTCCTCTGACTTCAACAGCG | |
| GAPDH (Reverse) | | ACCACCCTGTTGCTGTAGCCAA | |
|  | |  | |
| **Vector construction** | |  | |
| DTYMK cDNA (Forward) | | CGGGATCCATGGCGGCCCGGCGCGGGGC | |
| DTYMK cDNA (Reverse) | | CCCTCGAGTTACTTCCATAGCTCCCCCAG | |
| pGL6-miR DTYMK-wt (Forward) | | CGGGATCCCCCAAGGCTGCCCACTGGAGAC | |
| pGL6-miR DTYMK-wt (Reverse) | | CCCAAGCTTACTAGTGTCTGGAAGACATTTA | |
| pGL6-miR DTYMK-mut (Forward) | | GGCTGCCTCTTCTGCAGACAGACAGCATCTCACGCGG | |
| pGL6-miR DTYMK-mut (Reverse) | | CCACGGGGGTCCGCGTGAGATGCTGTCTGTCTGCAGAA | |
